# Chloroform Fatalities.—I

**Published:** 1895-06-15

**Authors:** Benjamin Ward Richardson


### Chloroform Fatalities.—I.

By Sir Benjamin Ward Richardson, M.D., F.R.S.

The chloroform fatalities have continued recently at a high rate, and have been rendered remarkable by the numbers of young subjects who have died during, or immediately after, administration. This last fact is particularly singular. In the years extending from 1864 to 1866, when I was writing the papers on the "Medical History of England," published in the *Medical Times and Gazette* of that day, I calculated the rate of mortality as it appeared in the records of various hospitals, and showed the figures to be one death in 2,500 cases, an estimate that has since been accepted as a standard. It was a curious circumstance that 17,000 administrations were noticed without the occurrence of a single death. Then came a death, then a second, until the proportion came to one in 2,500, which I believe at that time was truly correct. But the most singular fact of all is, that nearly all were deaths amongst adults. Scarce one child was included in the record. I find also, in perusing the late Dr. Snow's private record, that there was not an instance in his practice in which he so much as noticed a dangerous symptom in a child, and he expressed to me an opinion that he should never expect death to occur in a child from the administration of chloroform, because a child has not only special recuperative power, but because it is, like a lower animal, not under the influence of that fear of sudden death experienced by those of older and wiser growth. In these later days, however, the young share with the old in the fatal danger, and there must be some great cause somewhere for this increase of fatalities, some error in diagnosis, in purity of chloroform, in mode of administration, or some change in the susceptibilities of individuals operated upon. The suspicion rests on the mode of administration, and I am of opinion that this is the chief evil. There is nothing fixed in regard to administration, and if electricians or railway engineers were to proceed in the same irregular manner as we do in the administration of chloroform, the same irregular and bad results would inevitably ensue. The danger is excessive; the people are forced to become alive to the danger, and soon it will be a question at inquests whether the profession is sufficiently circumspect, or even competent, in the use it makes of the art of annulling pain. What then should be done?

There ought to be one uniform system of administration. Whilst one administrator is following one system, and another a different system, there can be no sound progress. The true advance lies in the introduction of a method that shall lead to the employment of the smallest possible amount of chloroform, in measured dose, in every instance.

There is very little doubt—none, I think—about Snow's estimate that eighteen minims of chloroform introduced into the blood is sufficient to produce the deepest anæsthesia that the surgeon can require. Let there be brought into practice therefore an inhaler that measures faithfully the quantity of the vapour; an inhaler as well attested for accuracy as a clinical thermometer is for determining temperature, and that shall be as universally applied. This is the first desideratum.

There should be greater care in selection of cases submitted to inhalation. Let the *ad captandum* plan of giving all who have to be operated on a general anæsthetic, be set aside, and in each instance let a more careful judgment be made. We bestow the utmost pains upon the mere operation, and if we think an operation likely to be fatal we very properly shun it; but we take no such strict precaution with regard to general anæsthesia, although it is certain that no person is ever rendered insensible without some actual risk to life. The chapter in Snow's book "On Alleged Cases of Death from Chloroform," is pregnant with fact on this point despite his ruling that whenever an operation is justifiable anæsthesia is justifiable. There are certain persons, say about one in 3,000, who at all times are ready to die. I have designated them the *morituri*, and these are the common victims of chloroform. We ought to be able to pick them out as cleverly as a shepherd picks out sickly and diseased sheep from his fold, deal with them according to our cultured judgment, refusing the dangerous cases when they are presented, at all risks of unpopularity, charges of timorousness, or any other reason. I know the difficulties, for I have withstood them. I remember well two distinguished surgeons looking at me with as much anger as surprise, because I once refused to administer chloroform to a lady who had large varicose veins upon which they were about to operate. I had found in her a feeble right heart with pallor (anæmia), a rather free obesity, and, as is usual in such persons, an extreme nervousness and fear of sudden death. She, I said to myself, belongs to the *morituri*, and I will take no part in the cause of her death. One of these surgeons, regardless of my caution, administered the chloroform himself, and saw the patient collapse into death after the first few inhalations from the flannel holding chloroform which he held over her face. The *morituri* should never take chloroform. They die from all shocks, and chloroform inhalation is as bad a shock as they can be subjected to. We would warn them from going up in a balloon; we subject them to a greater danger when we give them chloroform.

I am delicate in saying it, but I must tell what I sincerely believe, namely, that local anæsthesia, so popular for a time after the introduction of ether spray, is now far too much neglected. Surgeons tell me that there is an unwillingness to follow the local method, and that notwithstanding the improvements that have been made in compressing gases for spray purposes and the introduction of cocaine, the local anæsthesia is looked down upon and considered to be too troublesome to be instantly practicable. I am afraid there is some truth in this, and I am sure that certain of the fatal cases that have lately occurred would easily, safely, painlessly, and successfully have been subjected to the local method. I have many times myself applied the local method for the obvious *morituri*, thus saving them from all risk of death from a general anæsthetic. I never had reason to regret it.

